# Risk Assessment of Dietary Exposure to Organophosphorus Flame Retardants in Children by Using HBM-Data

**DOI:** 10.3390/toxics10050234

**Published:** 2022-05-03

**Authors:** Veronika Plichta, Johann Steinwider, Nina Vogel, Till Weber, Marike Kolossa-Gehring, Lubica Palkovičová Murínová, Soňa Wimmerová, Janja Snoj Tratnik, Milena Horvat, Gudrun Koppen, Eva Govarts, Liese Gilles, Laura Rodriguez Martin, Greet Schoeters, Adrian Covaci, Clémence Fillol, Loïc Rambaud, Tina Kold Jensen, Elke Rauscher-Gabernig

**Affiliations:** 1 Austrian Agency for Health and Food Safety(AGES), Division of Integrative Risk Assessment, Data & Statistics, Department of Risk Assessment, 1220 Vienna, Austria; johann.steinwider@ages.at (J.S.); elke.rauscher-gabernig@ages.at (E.R.-G.); 2 German Environment Agency (UBA), 06844 Dessau-Roßlau, Germany; nina.vogel@uba.de (N.V.); till.weber@uba.de (T.W.); marike.kolossa@uba.de (M.K.-G.); 3 Faculty of Public Health, Slovak Medical University, 833 03 Bratislava, Slovakia; lubica.murinova@szu.sk (L.P.M.); sona.wimmerova@szu.sk (S.W.); 4 Department of Environmental Sciences, Jožef Stefan Institute, 1000 Ljubljana, Slovenia; janja.tratnik@ijs.si (J.S.T.); milena.horvat@ijs.si (M.H.); 5 VITO Health, Flemish Institute for Technological Research (VITO), 2400 Mol, Belgium; gudrun.koppen@vito.be (G.K.); eva.govarts@vito.be (E.G.); liese.gilles@vito.be (L.G.); laura.rodriguezmartin@vito.be (L.R.M.); greet.schoeters@vito.be (G.S.); 6 Department of Biomedical Sciences, University of Antwerp, 2610 Wilrijk, Belgium; 7 Toxicological Center, University of Antwerp, 2610 Wilrijk, Belgium; adrian.covaci@uantwerpen.be; 8 Santé Publique France, French Public Health Agency (ANSP), 94415 Saint-Maurice, France; clemence.fillol@santepubliquefrance.fr (C.F.); loic.rambaud@santepubliquefrance.fr (L.R.); 9 Department of Environmental Medicine, Institute of Public Health, University of Southern Denmark, 5000 Odense, Denmark; tkjensen@health.sdu.dk

**Keywords:** HBM4EU, organophosphorus flame retardants, dietary exposure, children

## Abstract

Due to their extensive usage, organophosphorus flame retardants (OPFRs) have been detected in humans and in the environment. Human are exposed to OPFRs via inhalation of indoor air, dust uptake or dietary uptake through contaminated food and drinking water. Only recently, few studies addressing dietary exposure to OPFRs were published. In this study, we used human biomonitoring (HBM) data of OPFRs to estimate how much the dietary intake may contribute to the total exposure. We estimated by reverse dosimetry, the daily intake of tris (2-chloroethyl) phosphate (TCEP), tris (1-chloro-2-propyl) phosphate (TCIPP), tris (1,3-dichloro-2-propyl) phosphate (TDCIPP) for children using HBM data from studies with sampling sites in Belgium, Denmark, France, Germany, Slovenia and Slovakia. For estimating the dietary exposure, a deterministic approach was chosen. The occurrence data of selected food categories were used from a published Belgium food basket study. Since the occurrence data were left-censored, the Lower bound (LB)—Upper bound (UB) approach was used. The estimated daily intake (EDI) calculated on the basis of urine metabolite concentrations ranged from 0.03 to 0.18 µg/kg bw/d for TDCIPP, from 0.05 to 0.17 µg/kg bw/d for TCIPP and from 0.02 to 0.2 µg/kg bw/d for TCEP. Based on national food consumption data and occurrence data, the estimated dietary intake for TDCIPP ranged from 0.005 to 0.09 µg/kg bw/d, for TCIPP ranged from 0.037 to 0.2 µg/kg bw/d and for TCEP ranged from 0.007 to 0.018 µg/kg bw/d (summarized for all countries). The estimated dietary intake of TDCIPP contributes 11–173% to the EDI, depending on country and LB-UB scenario. The estimated dietary uptake of TCIPP was in all calculations, except in Belgium and France, above 100%. In the case of TCEP, it is assumed that the dietary intake ranges from 6 to 57%. The EDI and the estimated dietary intake contribute less than 3% to the reference dose (RfD). Therefore, the estimated exposure to OPFRs indicates a minimal health risk based on the current knowledge of available exposure, kinetic and toxicity data. We were able to show that the dietary exposure can have an impact on the general exposure based on our underlying exposure scenarios.

## 1. Introduction

Flame retardants are chemicals, which are added to materials to prevent and to delay the fire hazards. Due to their persistence and accumulation potency and because of toxicological concerns, brominated flame retardants e.g., polybrominated diphenyl ethers (PBDEs), such as penta-, octa-, and deca-BDE, and hexabromocyclododecanes (HBCDD) are regulated under the Stockholm Convention on Persistent Organic Pollutants. This has led to a surge in the usage of alternative flame retardants with similar technical characteristics, such as the organophosphorus flame retardants (OPFRs). OPFRs are not chemically bound to the product and can be released into the surroundings by leaching, evaporation, migration and abrasion from the polymer matrix during usage. Due to the extensive usage, OPFRs have been detected in humans and the environment. Humans are exposed to OPFRs via inhalative uptake especially in the indoor environment, and orally by uptake of dust or from the diet through contaminated food and drinking water [1,2,3]. In the human, OPFRs are rapidly absorbed and excreted via urine, the half-lives of OPFRs was assumed to be short (<1 day), but recent findings indicate a half-life of 3–53 days depending on the OPFRs [4]. Exposure to OPFRs may cause various adverse effects to the human body such as kidney toxicity, neurotoxicity, reproductive toxicity, carcinogenicity and endocrine disruption [5]. Within the European Union, some OPFRs are classified and labeled as Carc. 2 and, therefore, pose a health hazard [6]. In this study we focused on tris (2-chloroethyl) phosphate (TCEP), tris (1-chloro-2-propyl) phosphate (TCIPP) and tris (1,3-dichloro-2-propyl) phosphate (TDCIPP). TCIPP represents approximately 80% of the chlorinated OPFRs in Europe and is considered to be potentially carcinogenic. TCEP was replaced by TCIPP because of its lower toxicity. Studies indicate that TDCIPP might be more neurotoxic than TCEP and TCIPP [3]. Food can be contaminated with OPFRs by two routes. Firstly, crops and animal-derived and aquatic products can be contaminated with OPFRs via soil and water. Secondly, foodstuffs can be contaminated by OPFRs during the production process, packaging and storage, since OPFRs are also present in several materials used in food processing [6,7]. Human biomonitoring (HBM) studies, which address the exposure to OPFRs, had their focus mainly on the indoor environment as an exposure source, since OPFRs are detected in high amounts in indoor air and dust. Only recently, few studies focused on the dietary exposure to OPFRs [2,8,9,10] and concluded that the dietary exposure should be considered in future exposure assessments. Therefore, the aim of this risk assessment was to estimate the dietary exposure of TDCIPP, TCIPP, TCEP and their individual contribution to the general exposure. The latter was derived by using HBM data.

## 2. Materials and Methods

### 2.1. Human Biomonitoring Data

The used human biomonitoring data are part of the HBM4EU Aligned studies which have been described in detail by Gilles et al. [11]. Biobanked urine samples of children (6–12 years) from Belgium (3xG study), Denmark (OCC study), France (Esteban study), Germany (GerES V sub sample), Slovenia (SLO CRP study) and Slovakia (PCB cohort study) were collected and analysed for its bis (2-chloroethyl) phosphate (BCEP), bis (1-chloro-2-propyl) phosphate (BCIPP) and bis (1,3-dichloro-2-propyl) phosphate (BDCIPP) concentration to determine the exposure to TCEP, TCIPP and TDCIPP. A detailed description of the used HBM studies is given in the Supplementary Materials Table S1. In this risk assessment, when referring to a country, the HBM-study listed above is meant. However, the HBM- study does not always reflect the general exposure of the whole population of the country, since some of the studies were not population representative or only performed on a regional level.

#### Estimation of the Daily Intake (EDI)

The HBM data which were used for calculating the daily intake were not stratified and were volume based [µg/L] and non-creatinine adjusted. The estimated daily intake (*EDI*) from the urinary excretion of the OPFR was estimated by using the following equation:

$$EDI=\left(\frac{C_{Urine}*\;UV_{excr}}{Fue}\right)\times \left(\frac{MW_{d}}{MW_{m}}\right)$$

where *C_Urine_* is the concentration of the metabolite in µg/L and *UV_excr_* is the daily excreted urinary volume of 22.2 mL/kg bw/d for children [12]. *F_ue_* is the molar fraction of the urine excreted metabolite with respect to its parent compound, and *MW_d_* and *MW_m_* are the molecular weights of the parent compound and its metabolite. The following molecular weights were used [g/mol]: TCEP (285.49), BCEP (222.99), TCIPP (327.55), BCIPP (251.04), TDCIPP (430.9) and BDCIPP (319.93). For estimating the daily intake the arithmetic mean and the 95th Percentile (P95) of the urinary metabolite concentration were considered. The data on kinetics of the metabolism of OPFRs are limited, therefore the *F_ue_* of 0.63 for TDCIPP [13] was also used for TCIPP and TCEP. This equation is often used for substances with similar half–lives as e.g., for plasticizers [14].

### 2.2. Dietary Exposure

A deterministic approach was used to estimate the dietary intake of Belgian, Danish, French, German, Slovenian and Slovakian children. Since the toxicokinetic data are limited, it was assumed that 100% of the detected OPFRs concentration in the contaminated food is absorbed through the dietary intake. The provided occurrence data (see Table 1) originated from a Belgium food basket study published by Poma et al. [7], where samples were drawn from supermarkets, discount retailers and specialized meat and fish stores in two Belgian cities Brussels and Antwerp and in one village (Dessel) from spring 2015 to autumn 2016. In total, 165 samples belonging to 14 food categories were collected and analysed on its TDCIPP, TCIPP and TCEP concentration. The food category “animal and vegetable fats and oils and primary derivatives thereof” also included fish oil food supplement, which was the highest contaminated sample. This sample was excluded, since it is not regularly consumed by children and could distort the outcome. The edited occurrence data in regard to Lower Bound (LB), Medium Bound (MB) and Upper Bound (UB) were provided by Giulia Poma (2021; personal communication) and were not modified in this risk assessment. Since, the occurrence data were left-censored, the LB-, MB- and UB approach was used [15]. Occurrence data below the limit of quantification (LOQ) were set equal to zero (LB), to the half of the LOQ (MB) and equal to the LOQ (UB).

The used food consumption data, which were surveyed by food recording are given in Table 2. In general, the mean occurrence data were multiplied with the available mean food consumption data for children provided by the EFSA Comprehensive European Food Consumption Database [16]. This was conducted for the overall population (=all subjects) as well as for those who actually consumed the relevant food (=consumers) as reported in the respective national food consumption survey. Subsequently, the food category with the highest exposure of consumer was determined. For this food category, the P95 of the consumers and for the other food categories the mean of the overall population was used in order to calculate the exposure of high consumers. No food consumption data for Slovenian and Slovakian children were available, therefore the Austrian data were used, because these were the closest geographically data available.

### 2.3. Hazard and Risk Characterization

As no HBM4EU HBM-GV, neither a HBM-I nor a HBM-II value from the German Human biomonitoring Commission nor a biomonitoring equivalent (BE) were available, a health-based guidance value (HBGV) was derived on the basis of data for a toxicologically acceptable uptake. For each study population and exposure scenario (LB—UB), the estimated daily intake (EDI) and dietary intake were set in relation to the available HBGV which was derived from external uptake data. In the European Union Risk Assessment Report of TCIPP, a lowest observed adverse effect level (LOAEL) of 52 mg/kg bw/d was derived from a 13-week rat study. The LOAEL was based on the increased liver weight observed in male rats. A minimal margin of safety (MOS) for repeated dose toxicity of 50 was considered sufficient [22]. Furthermore, a reference dose (RfD) of 80 µg/kg bw/d was derived by Ali et al. [23], where the sensitive endpoint is unknown.

The Agency for Toxic Substances and Disease Registry (ATSDR) derived a minimal risk level (MRL) for TCEP of 0.2 mg/kg bw/d for chronic exposure (>365 days) based on renal tubule lesions in female rats [24]. The US-EPA derived a chronic provisional reference dose (p-RfD) of 0.007 mg/kg bw/d, based on an increased relative kidney weight (BMDL_1SD_ of 6.9 mg/kg bw/d) [25]. Another RfD of 22 µg/kg bw/d was derived by Ali et al. [23], where the sensitive endpoint is increased relative liver and kidney weights in female rats, as was reported by WHO [26]. For TDCIPP, an MRL of 0.2 mg/kg bw/d was derived for chronic exposure (>365 days) based on renal tubule lesions in male rats [24]. An RfD of 15 µg/kg bw/d was derived by Ali et al. [23], where the sensitive endpoint is increased liver weight in female mice, as was reported by WHO [26]. Additionally, a cumulative risk assessment by the use of the hazard index (HI) was performed for the urinary EDI and the estimated dietary exposure. The HI is calculated by summing up the hazard quotient (HQ) of the individual substances, whereby the HQ is the ratio of the HBGV and the estimated exposure of the respective substances. If the calculation yields a HI < 1, the combined risk is considered acceptable [27].

## 3. Results

### 3.1. Estimated Daily Intake (EDI)

The estimated daily intake (EDI) summarized for all countries (mean and high intake) ranged from 0.03 to 0.18 µg/kg bw/d for TDCIPP, from 0.05 to 0.17 µg/kg bw/d for TCIPP and from 0.02 to 0.2 µg/kg bw/d for TCEP. In Table 3, the urinary metabolites and the EDIs of their parent compounds are given in detail. BDCIPP had the highest detection frequency (df) across the studies (65–97.7%) except for Slovakia with 37% df. The df of BCIPP was below 55% across all studies, therefore only the P95 could be used. For BCEP the df ranged between 19 and 63.3%, so only for the German study was an arithmetic mean available and for Slovenia and Slovakia only the P95.

### 3.2. Dietary Exposure

TDCIPP was only detected in fish and cheese, whereas TCIPP and TCEP were present in almost all food groups [7].

**Figure 1:**
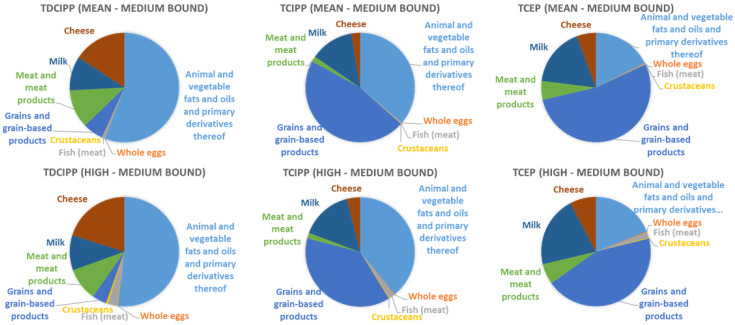
The contribution of the analysed food categories to the mean and high dietary exposure—an exemplary presentation of the medium bound.

The estimated dietary intake of TDCIPP ranged from 0.006 to 0.06 µg/kg bw/d (Belgium), 0.008 to 0.095 µg/kg bw/d (Denmark), 0.009 to 0.074 µg/kg bw/d (France), 0.005 to 0.06 µg/kg bw/d (Germany) and 0.008 to 0.08 µg/kg bw/d (Slovenia/Slovakia). For TCIPP the estimated dietary intake was 0.03 to 0.13 µg/kg bw/d for Belgium, 0.04 to 0.2 µg/kg bw/d for Denmark, 0.04 to 0.15 µg/kg bw/d for France, 0.04 to 0.14 µg/kg bw/d for Germany and 0.05 and 0.18 µg/kg bw/d for Slovenia/Slovakia. In regards to TCEP the dietary intake ranged from 0.008 and 0.018 µg/kg bw/d and 0.007 to 0.016 µg/kg bw/d for Slovenia/Slovakia and Germany respectively. As shown in Figure 1, food categories “animal and vegetable fats and oils and primary derivatives thereof”, “grains and grain-based products” “cheese” and “milk” were the main contributors to the dietary exposure. A detailed overview of the dietary intake is given in Supplementary Materials Tables S2–S13.

### 3.3. Risk Characterization

**Figure 2:**
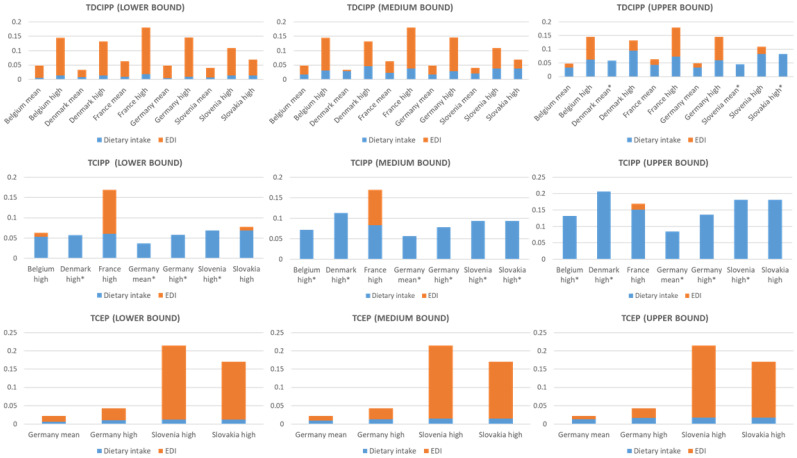
The contribution of the dietary exposure to the estimated daily intake in µg/kg bw/d. marked as * means the dietary intake exceeded the estimated daily intake, therefore, only the dietary intake is shown.

Figure 2 shows the dietary intake and its contribution to the EDI. In the calculated worst-case scenario (upper bound, high exposure), the estimated dietary intake of TDCIPP contributes 43% (Belgium), 72% (Denmark), 41% (France), 41% (Germany), 76% (Slovenia) and 120% (Slovakia). In the best-case scenario (lower bound, mean/high exposure) the estimated dietary intake contributes, 12% (Belgium), 25% (Denmark), 14% (France), 11% (Germany), 19% (Slovenia) and 20% (Slovakia, high exposure) to the EDI. The estimated dietary intake of TCIPP was in all scenarios above 100%, except for the Belgian (83%, scenario lower bound, high exposure) and the French (90%, scenario upper bound, high exposure) study populations. In the case of TCEP, it was estimated that the dietary intake ranges from 6 to 57% for the German, Slovenian and Slovakian study populations respectively.

Neither the EDI of TDCIPP and TCEP nor the estimated dietary exposure exceeded the RfD of 15 µg/kg bw/d or the p-RfD of 0.007 mg/kg bw/d. Its maximum contribution to the RfD is below 1.2% (TDCIPP) and 3% (TCEP). The EDI and dietary intake for TCIPP contributes only 0.01–0.21% to the RfD of 80 µg/kg bw/d. In the European Union Risk Assessment, a MOS approach for TCIPP was used, which we also considered. The estimated intake and the calculated dietary exposure were above the MOS of 50 (range 307,619–10,375,137; data not shown). The estimated HI is below 0.1. The EDI and dietary exposure of TDCIPP, TCIPP and TCEP are not likely to cause adverse health effects based on current knowledge from available exposure, kinetic and toxicity data. A detailed overview is given in Supplementary Materials Table S14.

In Table 4, the Risk Characterisation Ratio (RCR) was estimated with the used HBM data (P95) and the different published HBGVs. Although, the RCR is well below 1 and indicates no adverse health effects, the values differ depending on which HBGV was chosen. The available HBGVs ranged from 15–200 µg/kg bw/d for TDCIPP, from 80 to 52,000 µg/kg bw/d (LOAEL used for MOS) for TCIPP and from 7–200 µg/kg bw/d for TCEP. Anyways, it should be kept in mind, that in this risk assessment different HBGVs for one OPFR also might have different sensitive endpoints as it is the case for TDCIPP e.g., renal tubule lesions in male rats vs. increased liver weight in female mice. However, if more than one HBGV value is available, the lowest HBGV is normally chosen to conduct an evidence-based single substance risk assessment.

## 4. Discussion

### 4.1. Estimated Daily Intake

Only in the German study, BDCIPP, BCIPP and BCEP were detected frequently to estimate an average and high daily intake. The detected levels are in line with a previously published HBM study from Germany [8]. For the other HBM studies, no representative literature data were available to compare the detected levels in children within an acceptable sampling period. Anyways, it should also be considered, that only in the German, Slovenian, Belgian and French study population was the first morning urine sampled, this urine is normally more concentrated than spot urine. In a study conducted by Bastiaensen et al. [28], it was shown that the detection rate in a 24-h pooled urine sample is higher compared to spot urine samples. Furthermore, it was also shown that for certain OPFR metabolites such as BDCIPP, BCIPHIPP and DPHP, the first morning urine samples have the same representativeness as 24-h pooled samples. The authors concluded that the first morning urine samples might be a sensible alternative for biomonitoring studies to reduce exposure misclassification if the collection of several spot urine samples is not an option. The used metabolite might also have an impact on the EDI. The provided metabolite BDCIPP is known as an appropriate biomarker for TDCIPP since no other OPFR is metabolized to BDCIPP [29]. In the case of TCIPP, the provided metabolite BCIPP may not be the most representative biomarker. Several studies have reported that bis (1-chloro-2-propyl) 1-hydroxyl-2-propyl phosphate (BCIPHIPP) has a higher detection frequency than BCIPP and might be a more suitable biomarker to reflect the TCIPP body burden. Besides the biomarker BCEP, TCEP should also be considered as a biomarker, because of its resistance to be metabolized [30].

### 4.2. Dietary Intake

The estimated dietary intake of the sum of OPFRs ranged from 0.04 and 0.3 µg/kg bw/d (or 40–300 ng/kg bw/d), which is slightly lower compared to previous published data from Xu et al. [10]. They estimated a median dietary intake of 87 ng/kg bw/d and a high intake of 340 ng/kg bw/d of ∑OPFR (EHDPHP, TCEP, TPHP and TCPP) in a duplicate diet study. For the adult population, Poma et al. [2,7] estimated a mean dietary intake of 31 ng/kg bw/d (medium bound, Belgium food basket study) and mean dietary intake of 26.4 ng/kg bw/d (medium bound, Swedish food basket study). In all the above mentioned studies, EHDPHP was identified as the main contributor to the OPFR exposure. In this exposure assessment, the mean dietary intake (medium bound) was between 70 and 100 ng/kg bw/d for children.

The dietary exposure of TDCIPP contributes depending on the LB-MB-UB scenario between 7–173% to the EDI. In the case of TCEP, it is assumed that the dietary intake contributes 6–37% to the EDI. Except for France and the Belgian best case scenario (LB and high exposure), the estimated dietary exposure of TCIPP was in all scenarios above 100% to the EDI. In our study, the estimated total TCIPP intake in the German study was around 5 ng/kg bw/d. It was assumed that the total concentration in food was around 17.2 to 75.4 ng/g wet weight (ww; LB to UB), which might be an overestimation because it is unlikely that children in the used HBM studies consumed all the presented food categories in the time when the urine samples were collected. In a study performed by Xu et al. [10], they estimated that the total TCIPP exposure (inhalation, dust ingestion, dermal absorption and dietary intake) is around 8.6 ng/kg bw/d, which is comparable to our estimation. In their duplicate diet study, only 0.39 ng/g ww TCIPP was measured in the consumed food. In that study, air, dust and hand wipes were also analysed and based on the low concentration in food, they concluded the main exposure source was inhalation of personal ambient air [10,31]. Moreover, the provided biomarker might not reflect a realistic TCIPP exposure. This can also explain the higher estimated dietary exposure compared to the EDI.

The occurrence data were left-censored and for e.g., TDCIPP the detection frequency was quite low, it was only frequently detected in fish (df 39%) and cheese (df 44%). TCIPP and TCEP were more frequently detected in the range of 25–71% and 11–57% across the analysed food categories. In the LB-MB-UB approach, the food category “animal fats and vegetable fats and oils and primary derivatives of” contributes most to the total OPFRs exposure, which might not reflect a realistic exposure scenario because it was not detected at all. So it can either be not present in the samples or it was not detectable because of their high limit of quantification (LOQ) of 32.17 ng/g ww (TDCIPP) and of 61.97 ng/g ww (TCIPP) [7]. Based on the detection frequency of OPFRs in certain food categories, we conclude that a realistic exposure scenario occurs rather in the LB to MB range than in the UB. Despite the uncertainties by using aggregated occurrence data from a different country our estimations for the dietary intake in children are slightly higher than the data for adults in the literature. This is in line with the general observations that children have a higher uptake per kg body weight than adults due to their lower body weight.

Most of the published OPFRs human biomonitoring studies focused on the indoor exposure. The concentration in house dust is approximately three magnitudes higher than its occurrence in food. In contrast to this, a child ingests approximately 1.2 kg of food per day compared to 30 mg of dust [32]. Gbadamosi et al. [33] reviewed that for adults, food ingestion contributes 75%, 41% and 36% to the total TCEP, TDCIPP and TCIPP exposure. It can be assumed that the dietary intake might be equal or even a greater contributor to the total human exposure to OPFRs [6,7]. Similar to the few previous studies, we were able to show that the dietary exposure of OPFR can have an impact on the general exposure.

### 4.3. Uncertainties and Limitations

The following sources of uncertainties have been considered and are summarised in Table 5. Since we used the urinary molar excretion fraction (F_ue_) of TDCIPP also for TCIPP and TCEP, we are not able to determine if the EDIs are over- or underestimated. Further, we used non-creatinine adjusted urinary HMB-data, since we had no detailed information on the anthropometric parameters. Some studies indicated that creatinine adjustment is not the appropriate approach to address the urine dilution for some OPFR metabolites, since they can be conjugated in the liver as glucuronides or sulfates and be actively excreted by the renal tubes [28]. It shows that adjusting it to the specific gravity, the ratio between the density of the urine and pure water, is more accurate to use. Nevertheless, in this risk assessment we did not use adjusted urinary values, and do not believe that it has a high impact on the outcome but it is still an uncertainty that can cause an over- or underestimation of the EDI. The provided biomarker BCIPP might not reflect the TCIPP exposure appropriately because of its low detection frequency. Since we have no information on the urinary BCIPHIPP concentration, an underestimation of the TCIPP exposure is likely.

Based on the uncertainty of aggregated occurrence data and the low hierarchy in the food consumption data, the used approach is rather conservative and an overestimation of the dietary exposure is likely. Further the occurrence data originated from Belgium, which might not represent the contamination on a European level.

The toxicological data are limited so we are not able to estimate if the used HBGVs are leading to an over- or underestimation of the risk characterisation.

The limitations in this risk assessment and by using HBM data were the lack of information on toxicokinetics as urinary molar excretion fraction or the oral bioavailability, which is still assumed to be 100% and can result in an overestimation of the exposure or a health risk. All HBGV were published between 2009 and 2012, there are no new data available, which is also a limiting factor in the hazard assessment. Additionally, for TCIPP, Ali et al. [23] did not provide any further information from which toxicological endpoints the RfD was derived, it is only known that chronic NOAELs and an uncertainty factor of 1000 were used. Therefore, it is not possible to evaluate this RfD on its plausibility and values like this one should be used carefully.

A refinement on analytical methods to achieve more sensitive LOQ and LOD is necessary to estimate the contamination, the LOQ in the food category “fats and oils” was rather high. Therefore, we actually do not know if an OPFR contamination in this food category is likely or not. The same applies to the OPFRs metabolites, a more sensitive LOQ and LOD would lead to a more accurate detection frequency of metabolites and reflects a more realistic human body burden.

## 5. Conclusions

We were able to show that the dietary exposure can have an impact on the general exposure. Under consideration of the current knowledge the estimated daily intake and dietary exposure indicate a minimal health risk. Moreover, we showed how HBM data can be integrated into a dietary risk assessment without performing a duplicate diet study. This kind of approach is expensive and time intense and are mainly conducted on a regional level. Anyways, further research is needed to overcome the data limitations e.g., occurrence data to provide a more comprehensive picture of the OPFRs exposure in children. Uncertainties especially information on toxicokinetics as urinary molar excretion fraction or the oral bioavailability should be addressed and reduced. Further, it is recommended to refine the analytical methods to achieve more sensitive LOQ and LOD to estimate the contamination in food and body burden accurately.

## Figures and Tables

**Table 1:** Occurrence data of TDCIPP, TCIPP and TCEP concentration in the analysed food categories in µg/kg provided by Poma et al. (2018).

Food Category	TDCIPP				TCIPP				TCEP			
	df%	LB	MB	UB	df%	LB	MB	UB	df%	LB	MB	UB
Animal and vegetable fats and oils and primary derivatives thereof	0	5.7 *	16.1	34.7	0	11 *	31	66.8	11	1.55 *	2.5	3.6
Whole Eggs	0	0	0.05	0.1	25	0.1	0.15	0.19	25	0.02	0.03	0.04
Fish (meat)	39	0.4	0.5	0.6	59	0.71	0.76	0.8	54	0.12	0.13	0.14
Crustaceans	0	0	0.22	0.44	20	0.18	0.22	0.26	0	0	0.04	0.07
Grains and grain-based products	0	0	0.16	0.31	71	3.58	3.65	3.73	57	0.6	0.61	0.63
Meat and meat products	0	0	0.8	1.61	26	0.19	0.29	0.39	34	0.15	0.2	0.25
Milk	0	0	0.21	0.43	0	0	0.8	1.59	0	0	0.23	0.45
Cheese	44	2.52	3.09	3.65	50	1.42	1.53	1.63	50	0.66	0.71	0.77

df: detection frequency [%], LB: Lower bound, MB: Medium bound, UB: Upper bound, * the LB data were provided by G. Poma (personal communication).

**Table 2:** Food consumption data presented by country and food groups [g/kg bw/d] (EFSA, 2021).

Food Category	Consumption Data Germany ^1^		Consumption Data Austria ^2,^ *		Consumption Data Belgium ^3^		Consumption Data Denmark ^4^		Consumption Data France ^5^	
	Total Mean Population	High Level Consumer Only	Total Mean Population	High Level Consumer Only	Total Mean Population	High Level Consumer Only	Total Mean Population	High Level Consumer Only	Total Mean Population	High Level Consumer Only
Animal and vegetable fats and oils and primary derivatives thereof	0.58	1.34	0.92	1.98	0.48	1.29	1.14	2.2	0.62	1.5
Whole Eggs	0.67	2.15	0.47	1.59	0.01	1.47	0.5	1.44	0.05	0.89
Fish (meat)	0.21	2.59	0.38	2.62	0.35	4.36	0.4	1.52	0.54	4.01
Crustaceans	0.0004	1	0.01	1.54	0.05	2.52	0.03	0.43	0.04	1.82
Grains and grain-based products	8.16	14.03	9.4	15.76	7.31	12.65	7.72	11.85	8.04	13.97
Meat and meat products	2.75	6.01	2.86	6.67	4.14	9.17	3.83	6.6	4.38	9.14
Milk	8.46	20.98	6.68	16.54	9.16	27.11	17.26	35.65	10	26.69
Cheese	0.74	2.28	0.89	2.25	1.09	4.37	0.73	1.88	2.05	6.07

^1^ national food consumption survey from 2006 [17], ^2^ national food consumption survey from 2010, * used for Slovenia and Slovakia [18] ^3^ national food consumption survey from 2014 [19] ^4^ national food consumption survey from 2005 [20] and ^5^ national food consumption survey from 2014 [21].

**Table 3:** Urinary BDCIPP, BCIPP and BCEP concentration levels and the estimated daily intakes (EDI) of their parent compounds TDCIPP, TCIPP and TCEP.

Country	OPFR-Metabolite	n	Df	Urinary Concentration [µg/L]		EDI of Parent Compound	
			[%]			[µg/kg bw/d]	
				Mean	High	Mean	High
Belgium (3xG study)	BDCIPP	133	98	1.03	3.08	0.05	0.14
	BCIPP	133	13.5	-	1.4	-	0.063
Denmark (OCC study)	BDCIPP	291	97	0.72	2.8	0.03	0.13
	BCIPP	291	6.5	-	0.48		0.022
France (Esteban study)	BDCIPP	299	65	1.34	3.82	0.06	0.18
	BCIPP	299	31	-	3.71		0.17
Germany (GerES V study)	BDCIPP	300	80	1.03	3.09	0.05	0.15
	BCIPP	300	53	0.11 ^1^	0.74	0.05	0.034
	BCEP	300	63	0.48	0.96	0.022	0.043
Slovenia (SLO CRP study)	BDCIPP	147	84	0.86	2.32	0.04	0.11
	BCIPP	147	18	-	0.6	-	0.027
	BCEP	147	20	-	4.79	-	0.214
Slovakia (PCB cohort study)	BDCIPP	300	17	-	1.46	-	0.07
	BCIPP	300	29	-	1.71	-	0.078
	BCEP	300	20	-	3.81	-	0.17

df: detection frequency in percent, arithmetic mean and high (P95), ^1^ the median has been used, since no arithmetic mean was available.

**Table 4:** Risk Characterisation Ratio (RCR) of the used HBM data (P95) with the different HBGVs and the estimation of the Margin of Safety.

	TDCIPP		TCIPP		TCEP		
	MRL	RfD	RfD	MOS *	MRL	p-RfD	RfD
	(200 µg/kg bw/d)	(15 µg/kg bw/d)	(80 µg/kg bw/d)	(52 mg/kg bw/d)	(200 µg/kg bw/d)	(7 µg/kg bw/d)	(22 µg/kg bw/d)
Belgium	0.001	0.01	0.001	823,423	-	-	-
Denmark	0.001	0.01	0.0003	2,360,912	-	-	-
France	0.0009	0.01	0.002	307,619	-	-	-
Germany	0.0007	0.01	0.0004	1,538,093	0.0002	0.006	0.002
Slovenia	0.00054	0.0073	0.0003	1,908,470	0.001	0.03	0.01
Slovakia	0.0003	0.004	0.001	667,016	0.001	0.024	0.008

* Margin of Safety (MOS) is the ratio of NOAEL (or LOAEL) obtained from animal toxicological studies to the predicted or estimated human exposure. In this case a MOS over 50 is considered to be protective.

**Table 5:** Qualitative evaluation of influence of uncertainties on the dietary exposure estimate and the estimated daily intake.

Sources of Uncertainty	Direction
**Dietary exposure estimates**	
The use of aggregated occurrence and food consumption data	+
**Occurrence data**	
The used occurrence data were from a single study and from only one country	+/−
Concentration data are considered applicable for all items within the entire food category	+
**Food consumption data**	
The used food consumption data were at a low hierarchy level	+
The used food consumption data were collected between 2006–2014 and might be outdated	+/−
For Slovenia and Slovakia no food consumption data were available, data from the geographically closest region was used	+/−
Use of data from food consumption surveys of a few days to estimate long-term (chronic) exposure for high percentiles (95th percentiles)	+
**Hazard data**	
Ali et al., 2012 did not provide information on the toxicological study and endpoints from which the reference dose for TCIPP was derived	+/−
Health-Based Guidance Values are only based on limited toxicological data	+/−
Uncertainty factors ranged from 100 to 1000	+/−
**Estimated daily intake by using HBM data**	
Extrapolation from single non-creatinine adjusted urine sample (spot or morning urine sample) to a 24 h urine sample	+/−
Slovakia and Denmark provided spot urine samples (less concentrated as first morning urine)	-
The estimated urinary molar excretion fraction (F_ue_) from TDCIPP was used for TCIPP and TCEP	+/−
Absence of individual urine excretion volume data	+/−
BCIPP was the only provided biomarker for TCIPP	-

+: uncertainty with potential to cause overestimation of exposure; -: uncertainty with potential to cause underestimation of exposure; +/−: uncertainty can cause either an over- or underestimation of exposure.

## Data Availability

Not applicable.

## Supplementary Materials

The following supporting information can be downloaded at: https://www.mdpi.com/article/10.3390/toxics10050234/s1, Table S1. Detailed description of the used HBM studies, Table S2. Estimated dietary TDCIPP ex-posure for children in Slovenia and Slovakia (µg/kg bw/day), Table S3. Estimated dietary TCIPP exposure for children in Slovenia and Slovakia (µg/kg bw/day), Table S4. Estimated dietary TCEP exposure for children in Slovenia and Slovakia (µg/kg bw/day), Table S5. Estimated die-tary TDCIPP exposure for children in Germany (µg/kg bw/day), Table S6. Estimated dietary TCIPP exposure for children in Germany (µg/kg bw/day), Table S7. Estimated dietary TCEP ex-posure for children in Germany (µg/kg bw/day), Table S8. Estimated dietary TDCIPP exposure for children in Belgium (µg/kg bw/day), Table S9. Estimated dietary TCIPP exposure for children in Belgium (µg/kg bw/day), Table S10. Estimated dietary TDCIPP exposure for children in Den-mark (µg/kg bw/day), Table S11. Estimated dietary TCIPP exposure for children in Denmark (µg/kg bw/day), Table S12. Estimated dietary TDCIPP exposure for children in France (µg/kg bw/day), Table S13. Estimated dietary TCIPP exposure for children in France (µg/kg bw/day), Table S14. Dietary exposure estimation from each cohort in µg/kg bw/d and its contribution to the estimated intake and its relation to the RfD of 15 µg/kg bw/d (for TDCIPP) or 80 µg/kg bw/d (for TCIPP) and a p-RfD of 7 µg/kg bw/d (for TCEP) in percent.
