# Supplementary material for: Risk Assessment of Dietary Exposure to Organophosphorus Flame Retardants in Children by Using HBM-Data
Source: Toxics. 2022 May 3;10(5):234. doi: 10.3390/toxics10050234 (PMC9144966; doi:10.3390/toxics10050234)
Supplement: Supplementary file 1 [file toxics-10-00234-s001.zip › toxics-1685752-supplementary.pdf]

toxics MDPI

Supplementary Materials: Risk Assessment of Dietary Exposure to Organophosphorus Flame Retardants in Children by Using HBM-Data

Veronika Plichta, Johann Steinwider, Nina Vogel, Till Weber, Marike Kolossa-Gehring, Lubica Palkovičová Murínová, Soňa Wimmerová, Janja Snoj Tratnik, Milena Horvat, Gudrun Koppen, Eva Govarts, Liese Gilles, Laura Rodriguez Martin, Greet Schoeters, Adrian Covaci, Clémence Fillol, Loïc Rambaud, Tina Kold Jensen and Elke Rauscher-Gabernig

Table S1. Detailed description of the used HBM studies.

HBM study	Country/ EU Region	Age in years	Sampling periode	Urine sample	Sex (%)	Residential degree (%)
3xG Study	Belgium (EU west)	6-8	2019-2020	First morning urine	50 (f) 50 (m)	Towns/Suburbs (100)
OCC Study	Denmark (EU north)	6-7	2018-2019	Spot urine	45 (f) 55 (m)	Cities (100)
Esteban study	France (EU west)	6-12	2014-2016	First morning urine	46 (f) 54 (m)	Rural areas (48) Towns/Suburbs (27) Cities (25)
GerES V sub-study	Germany (EU west)	6-12	2015-2018	First morning urine	50 (f) 50 (m)	Towns/Suburbs (44) Rural areas (34) Cities (22)
SLO CRP study	Slovenia (EU south)	7-10	2018	First morning urine	55 (f) 45 (m)	Rural areas (100)
PCB cohort study	Slovakia (EU east)	10-12	2014-2017	Spot urine	56 (f) 44 (m)	Rural areas (54) Towns/Suburbs (46)

Table S2. Estimated dietary TDCIPP exposure for children in Slovenia and Slovakia (µg/kg bw/day).

	Lower bound		Medium Bound		Upper Bound	
	mean	high	mean	high	mean	high
Animal and vegetable fats and oils and primary derivatives thereof	0.01	0.01	0.01	0.032	0.032	0.069
Whole Eggs	0	0	0.0002	0.0001	0.00005	0.0002
Fish (meat)	0.00015	0.0010	0.0002	0.001	0.0002	0.0016
Crustaceans	0.00	0.0000	0.000001	0.0003	0.000002	0.0007
Grains and grain-based products	0.00	0.0000	0.002	0.0025	0.003	0.005
Meat and meat products	0	0	0.0002	0.0005	0.005	0.011

Milk	0	0	0.001	0.0035	0.003	0.007
Cheese	0.0022	0.0057	0.003	0.007	0.003	0.008
**Total exposure**	**0.008**	**0.014**	**0.02**	**0.038**	**0.05**	**0.083**

Table S3. Estimated dietary TCIPP exposure for children in Slovenia and Slovakia (μg/kg bw/day).

	**Lower bound**		**Medium Bound**		**Upper Bound**	
	mean	high	mean	high	mean	high
Animal and vegetable fats and oils and primary derivatives thereof	0.01	0.0218	0.0284	0.0614	0.06	0.132
Whole Eggs	0.00005	0.0002	0.0001	0.0002	0.00009	0.0003
Fish (meat)	0.0003	0.0019	0.0003	0.0020	0.0003	0.0021
Crustaceans	0.000001	0.0003	0.000001	0.0003	0.000001	0.0004
Grains and grain-based products	0.03	0.0564	0.0343	0.0575	0.0351	0.059
Meat and meat products	0.0005	0.0013	0.0008	0.0019	0.0011	0.0026
Milk	0	0	0.0053	0.0132	0.0106	0.026
Cheese	0.0013	0.0032	0.0014	0.0034	0.0014	0.004
**Total exposure**	**0.05**	**0.07**	**0.071**	**0.094**	**0.11**	**0.181**

Table S4. Estimated dietary TCEP exposure for children in Slovenia and Slovakia (μg/kg bw/day).

	**Lower bound**		**Medium Bound**		**Upper Bound**	
	mean	high	mean	high	mean	high
Animal and vegetable fats and oils and primary derivatives thereof	0.001	0.0031	0.0022	0.0047	0.0033	0.0071
Whole Eggs	0.000009	0.00003	0.00001	0.00005	0.00002	0.0001
Fish (meat)	0.00005	0.0003	0.00005	0.0003	0.0001	0.0004
Crustaceans	0.00	0.00	0.0000002	0.0001	0.0000004	0.0001
Grains and grain-based products	0.006	0.0095	0.0057	0.0096	0.0059	0.01
Meat and meat products	0.0004	0.0010	0.0006	0.0013	0.0007	0.0017
Milk	0	0	0.0015	0.0038	0.003	0.0074
Cheese	0.0006	0.0015	0.0006	0.0016	0.0007	0.0017

**Total exposure**	**0.008**	**0.012**	**0.011**	**0.015**	**0.014**	**0.018**

Table S5. Estimated dietary TDCIPP exposure for children in Germany (µg/kg bw/day).

	**Lower bound**		**Medium Bound**		**Upper Bound**	
	mean	high	mean	high	mean	high
Animal and vegetable fats and oils and primary derivatives thereof	0.0033	0.0076	0.0093	0.0215	0.0200	0.046
Whole Eggs	0	0	0.00003	0.0001	0.0001	0.0002
Fish (meat)	0.00009	0.0010	0.0001	0.0013	0.0001	0.0016
Crustaceans	0	0	0.000001	0.0002	0.000002	0.0004
Grains and grain-based products	0	0	0.0013	0.0022	0.0025	0.0043
Meat and meat products	0	0	0.0022	0.0048	0.0044	0.0097
Milk	0	0	0.0018	0.0044	0.0036	0.009
Cheese	0.0019	0.0057	0.0023	0.0070	0.0027	0.0083
**Total exposure**	**0.005**	**0.010**	**0.017**	**0.029**	**0.03**	**0.06**

Table S6. Estimated dietary TCIPP exposure for children in Germany (µg/kg bw/day).

	**Lower bound**		**Medium Bound**		**Upper Bound**	
	mean	high	mean	high	mean	high
Animal and vegetable fats and oils and primary derivatives thereof	0.01	0.0147	0.0179	0.0415	0.04	0.0893
Whole Eggs	0.00007	0.0002	0.0001	0.0003	0.00013	0.0004
Fish (meat)	0.00015	0.0018	0.0002	0.002	0.00017	0.0021
Crustaceans	0.0000007	0.0002	0.000001	0.0002	0.000001	0.0003
Grains and grain-based products	0.029	0.0502	0.03	0.051	0.03	0.052
Meat and meat products	0.0005	0.0011	0.0008	0.0017	0.0011	0.0023
Milk	0	0	0.007	0.017	0.013	0.033
Cheese	0.0010	0.0032	0.0011	0.0035	0.0012	0.0037
**Total exposure**	**0.037**	**0.058**	**0.06**	**0.08**	**0.085**	**0.136**

Table S7. Estimated dietary TCEP exposure for children in Germany (µg/kg bw/day).

	**Lower bound**		**Medium Bound**		**Upper Bound**	
	mean	high	mean	high	mean	high
Animal and vegetable fats and oils and primary derivatives thereof	0	0.0021	0.0014	0.0032	0.0021	0.0048
Whole Eggs	0.000013	0.0000	0.00002	0.0001	0.00003	0.0001
Fish (meat)	0.00003	0.0003	0.00003	0.0003	0.00003	0.0004
Crustaceans	0.00	0.00	0.0000001	0.00004	0.0000003	0.0001
Grains and grain-based products	0.005	0.0084	0.0050	0.0086	0.0051	0.0088
Meat and meat products	0.0004	0.0009	0.0005	0.0012	0.0007	0.0015
Milk	0	0	0.0019	0.0048	0.0038	0.0094
Cheese	0.0005	0.0015	0.0005	0.0016	0.0006	0.0018
**Total exposure**	**0.007**	**0.010**	**0.009**	**0.013**	**0.0123**	**0.0160**

Table S8. Estimated dietary TDCIPP exposure for children in Belgium (µg/kg bw/day).

	**Lower bound**		**Medium Bound**		**Upper Bound**	
	mean	high	mean	high	mean	high
Animal and vegetable fats and oils and primary derivatives thereof	0.0027	0.0074	0.0077	0.0208	0.016	0.045
Whole Eggs	0	0	0.000001	0.0001	0.000001	0.0001
Fish (meat)	0.00014	0.0017	0.0002	0.0022	0.0002	0.0026
Crustaceans	0	0	0.000011	0.0006	0.000022	0.0011
Grains and grain-based products	0	0	0.0012	0.002	0.0023	0.0039
Meat and meat products	0	0	0.0033	0.007	0.0067	0.015
Milk	0	0	0.002	0.0057	0.004	0.01
Cheese	0.0027	0.01	0.0034	0.013	0.004	0.016
**Total exposure**	**0.006**	**0.01**	**0.018**	**0.031**	**0.03**	**0.06**

Table S9. Estimated dietary TCIPP exposure for children in Belgium (µg/kg bw/day).

	**Lower bound**		**Medium Bound**		**Upper Bound**	
	mean	high	mean	high	mean	high
Animal and vegetable fats and oils and primary derivatives thereof	0.01	0.014	0.014	0.04	0.03	0.086
Whole Eggs	0.000001	0.0001	0.000002	0.0002	0.000002	0.003
Fish (meat)	0.00025	0.0031	0.003	0.0033	0.00028	0.0035
Crustaceans	0.0000090	0.0005	0.000011	0.0006	0.000013	0.0007
Grains and grain-based products	0.026	0.045	0.027	0.046	0.03	0.047
Meat and meat products	0.0008	0.0017	0.0012	0.0027	0.0016	0.0036
Milk	0	0	0.0073	0.022	0.015	0.043
Cheese	0.0015	0.0062	0.0017	0.0067	0.0018	0.0071
**Total exposure**	**0.034**	**0.053**	**0.052**	**0.072**	**0.078**	**0.132**

Table S10. Estimated dietary TDCIPP exposure for children in Denmark (µg/kg bw/day).

	**Lower bound**		**Medium Bound**		**Upper Bound**	
	mean	high	mean	high	mean	high
Animal and vegetable fats and oils and primary derivatives thereof	0.0065	0.0125	0.0183	0.0354	0.0395	0.0763
Whole Eggs	0	0	0.000025	0.0001	0.000050	0.0001
Fish (meat)	0.00016	0.0006	0.0002	0.0008	0.0002	0.0009
Crustaceans	0	0	0.000007	0.0001	0.000013	0.0002
Grains and grain-based products	0	0	0.0012	0.0019	0.0024	0.0037
Meat and meat products	0	0	0.0031	0.0053	0.0062	0.0106
Milk	0	0	0.0036	0.0075	0.0074	0.0153
Cheese	0.0018	0.0047	0.0023	0.0058	0.0027	0.0069
**Total exposure**	**0.008**	**0.015**	**0.029**	**0.046**	**0.06**	**0.095**

Table S11. Estimated dietary TCIPP exposure for children in Denmark (μg/kg bw/day).

	**Lower bound**		**Medium Bound**		**Upper Bound**	
	mean	high	mean	high	mean	high
Animal and vegetable fats and oils and primary derivatives thereof	0.01	0.0242	0.0353	0.0682	0.08	0.1469
Whole Eggs	0.000050	0.0001	0.000075	0.0002	0.000095	0.0003
Fish (meat)	0.00028	0.0011	0.0003	0.0012	0.00032	0.0012
Crustaceans	0.0000054	0.0001	0.000007	0.0001	0.000008	0.0001
Grains and grain-based products	0.028	0.0424	0.0282	0.0433	0.03	0.044
Meat and meat products	0.0007	0.0013	0.0011	0.0019	0.0015	0.0026
Milk	0	0	0.0138	0.0285	0.027	0.057
Cheese	0.0010	0.0027	0.0011	0.0029	0.0012	0.0031
**Total exposure**	**0.042**	**0.057**	**0.080**	**0.113**	**0.135**	**0.206**

Table S12. Estimated dietary TDCIPP exposure for children in France (μg/kg bw/day).

	**Lower bound**		**Medium Bound**		**Upper Bound**	
	mean	high	mean	high	mean	high
Animal and vegetable fats and oils and primary derivatives thereof	0.0035	0.0086	0.0100	0.0241	0.0215	0.0520
Whole Eggs	0	0	0.000003	0.00004	0.000005	0.0001
Fish (meat)	0.00022	0.0016	0.0003	0.0020	0.0003	0.0024
Crustaceans	0	0	0.000009	0.0004	0.000018	0.0008
Grains and grain-based products	0	0	0.0013	0.0022	0.0025	0.0043
Meat and meat products	0	0	0.0035	0.0073	0.0071	0.0147
Milk	0	0	0.0021	0.0056	0.0043	0.0115
Cheese	0.0052	0.0153	0.0063	0.0188	0.0075	0.0222
**Total exposure**	**0.009**	**0.019**	**0.023**	**0.038**	**0.04**	**0.074**

Table S13. Estimated dietary TCIPP exposure for children in France (μg/kg bw/day).

	Lower bound		Medium Bound		Upper Bound	
	mean	high	mean	high	mean	high
Animal and vegetable fats and oils and primary derivatives thereof	0.007	0.0165	0.0192	0.0465	0.04	0.1001
Whole Eggs	0.000005	0.0001	0.000008	0.0001	0.00001	0.0002
Fish (meat)	0.00038	0.0028	0.0004	0.0030	0.00043	0.0032
Crustaceans	0.000007	0.0003	0.000009	0.0004	0.00001	0.0005
Grains and grain-based products	0.029	0.0500	0.0293	0.0510	0.03	0.052
Meat and meat products	0.0008	0.0017	0.0013	0.0027	0.0017	0.0036
Milk	0	0	0.0080	0.0214	0.016	0.042
Cheese	0.0029	0.0086	0.0031	0.0093	0.0033	0.0099
**Total exposure**	**0.040**	**0.061**	**0.061**	**0.083**	**0.093**	**0.152**

Table S14. Dietary exposure estimation from each cohort in μg/kg bw/d and its contribution to the estimated intake and its relation to the RfD of 15 μg/kg bw/d (for TDCIPP) or 80 μg/kg bw/d (for TCIPP) and a p-RfD of 7 μg/kg bw/d (for TCEP) in percent.

	Estimated daily intake				Dietary intake lower bound						Dietary intake medium bound						Dietary intake upper bound					
	mean	% of RfD	high	% of RfD	mean	% of EDI	% of RfD	high	% of EDI	% of RfD	mean	% of EDI	%of RfD	high	% of EDI	%of RfD	mean	% of EDI	% of RfD	high	% of EDI	% of RfD
**Belgium**																						
**TDCIPP**	0.05	0.3	0.15	1	0.006	12	0.04	0.01	7	0.09	0.018	37	0.12	0.031	21	0.2	0.03	70	0.22	0.06	43	0.41
**TCIPP**	-	-	0.06	0.08	0.034	-	0.04	0.05	84	0.07	0.05	-	0.07	0.072	113	0.09	0.07	-	0.1	0.13	208	0.16
**∑OPFR**	-	-	0.21		0.04			0.06			0.07			0.103			0.1			0.2		
**Hazard Index**			0.01		0.0008			0.0016			0.002			0.003			0.003			0.006		
**Denmark**																						
**TDCIPP**	0.03	0.2	0.13	0.9	0.008	25	0.06	0.015	11	0.1	0.029	85	0.2	0.046	35	0.3	0.06	173	0.4	0.09	72	0.6
**TCIPP**	-	-	0.02	0.03	0.042	-	0.05	0.057	259	0.07	0.08	-	0.1	0.113	512	0.14	0.13		0.17	0.21	936	0.26
**∑OPFR**	-	-	0.15		0.05			0.07			0.11			0.16			0.19			0.3		
**Hazard Index**			0.009		0.001			0.002			0.003			0.004			0.006			0.009		
**France**																						
**TDCIPP**	0.06	0.4	0.18	1.2	0.009	14	0.06	0.019	11	0.13	0.02	37	0.16	0.04	21	0.25	0.04	68	0.3	0.07	41	0.5
**TCIPP**	-	-	0.17	0.2	0.04		0.05	0.06	36	0.08	0.06		0.08	0.08	49	0.1	0.09		0.12	0.15	90	0.19
**∑OPFR**			0.35		0.05			0.08			0.085			0.121			0.14			0.225		
**Hazard Index**			0.01		0.001			0.002			0.002			0.003			0.004			0.007		
**Germany**																						
**TDCIPP**	0.05	0.3	0.15	1	0.005	11	0.03	0.01	7	0.06	0.017	35	0.01	0.03	20	0.2	0.03	69	0.22	0.06	41	0.4
**TCIPP**	0.005	0.01	0.03	0.04	0.04	745	0.05	0.06	173	0.07	0.06	1130	0.07	0.08	231	0.10	0.08	1696	0.11	0.14	402	0.17
**TCEP**	0.02	0.3	0.04	0.6	0.007	31	0.1	0.01	24	0.15	0.01	43	0.13	0.01	30	0.19	0.01	57	0.18	0.01	37	0.23
**∑OPFR**	0.075		0.22		0.05			0.08			0.08			0.12			0.13			0.21		
**Hazard Index**	0.006		0.016		0.002			0.003			0.003			0.005			0.005			0.008		
**Slovenia**																						
**TDCIPP**	0.04	0.3	0.11	0.7	0.008	19	0.05	0.014	13	0.09	0.02	52	0.14	0.04	35	0.25	0.05	113	0.3	0.08	76	0.5
**TCIPP**	-		0.03	0.03	0.05	-	0.06	0.07	252	0.09	0.07	-	0.09	0.09	344	0.12	0.1	-	0.14	0.18	664	0.23

**TCEP**	-	0.21	3	0.008	-	0.12	0.012	6	0.17	0.01	-	0.15	0.015	7	0.21	0.014	-	0.2	0.018	8	0.25
**∑OPFR**	-	0.35		0.06			0.09			0.1			0.15			0.17			0.28		
**Hazard Index**		0.04		0.002			0.003			0.004			0.006			0.006			0.01		
**Slovakia**																					
**TDCIPP**	-	0.07	0.46	0.008	-	0.05	0.014	20	0.09	0.02	-	0.14	0.04	55	0.25	0.05	-	0.3	0.08	120	0.5
**TCIPP**	-	0.08	0.10	0.05	-	0.06	0.07	84	0.09	0.07	-	0.09	0.09	120	0.12	0.1	-	0.14	0.18	232	0.23
**TCEP**	-	0.17	2	0.008	-	0.12	0.012	7	0.17	0.01	-	0.15	0.015	9	0.21	0.014	-	0.2	0.018	10	0.25
**∑OPFR**		0.31		0.06			0.09			0.1			0.15			0.17			0.28		
**Hazard Index**		0.03		0.002			0.003			0.004			0.006			0.006			0.01		
